# LIRRN: Location-Independent Relative Radiometric Normalization of Bitemporal Remote-Sensing Images

**DOI:** 10.3390/s24072272

**Published:** 2024-04-02

**Authors:** Armin Moghimi, Vahid Sadeghi, Amin Mohsenifar, Turgay Celik, Ali Mohammadzadeh

**Affiliations:** 1 Ludwig-Franzius Institute of Hydraulic, Estuarine and Coastal Engineering, Leibniz University Hannover, Nienburger Str. 4, 30167 Hannover, Germany; 2 Department of Photogrammetry and Remote Sensing, K. N. Toosi University of Technology, Tehran 19967-15433, Iran; a.mohsenifar@email.kntu.ac.ir (A.M.); a_mohammadzadeh@kntu.ac.ir (A.M.); 3 Department of Geomatics, Faculty of Civil Engineering, University of Tabriz, Tabriz 51666-16471, Iran; v.sadeghi@tabrizu.ac.ir; 4 School of Electrical and Information Engineering, University of the Witwatersrand, Johannesburg 2000, South Africa; celikturgay@gmail.com; 5 Wits Institute of Data Science, University of the Witwatersrand, Johannesburg 2000, South Africa; 6 Faculty of Engineering and Science, University of Agder, 4604 Kristiansand, Norway

**Keywords:** relative radiometric normalization (RRN), location-independent RRN, remote sensing (RS), pseudo-invariant features (PIFs), bitemporal multispectral images, change detection

## Abstract

Relative radiometric normalization (RRN) is a critical pre-processing step that enables accurate comparisons of multitemporal remote-sensing (RS) images through unsupervised change detection. Although existing RRN methods generally have promising results in most cases, their effectiveness depends on specific conditions, especially in scenarios with land cover/land use (LULC) in image pairs in different locations. These methods often overlook these complexities, potentially introducing biases to RRN results, mainly because of the use of spatially aligned pseudo-invariant features (PIFs) for modeling. To address this, we introduce a location-independent RRN (LIRRN) method in this study that can automatically identify non-spatially matched PIFs based on brightness characteristics. Additionally, as a fast and coregistration-free model, LIRRN complements keypoint-based RRN for more accurate results in applications where coregistration is crucial. The LIRRN process starts with segmenting reference and subject images into dark, gray, and bright zones using the multi-Otsu threshold technique. PIFs are then efficiently extracted from each zone using nearest-distance-based image content matching without any spatial constraints. These PIFs construct a linear model during subject–image calibration on a band-by-band basis. The performance evaluation involved tests on five registered/unregistered bitemporal satellite images, comparing results from three conventional methods: histogram matching (HM), blockwise KAZE, and keypoint-based RRN algorithms. Experimental results consistently demonstrated LIRRN’s superior performance, particularly in handling unregistered datasets. LIRRN also exhibited faster execution times than blockwise KAZE and keypoint-based approaches while yielding results comparable to those of HM in estimating normalization coefficients. Combining LIRRN and keypoint-based RRN models resulted in even more accurate and reliable results, albeit with a slight lengthening of the computational time. To investigate and further develop LIRRN, its code, and some sample datasets are available at link in Data Availability Statement.

## 1. Introduction

Relative radiometric normalization (RRN) is the key step for justifying radiometric distortions between bi/multitemporal remote-sensing (RS) images caused by diverse atmospheric interferences, fluctuations in the sun–target–sensor geometry, and sensor characteristics [1,2,3,4]. The RRN technique’s aim is to find pixel pairs between reference and subject images and radiometrically align them through a linear/nonlinear mapping function (MF) [5]. Based on the selection criteria for these pairs, RRN methods are broadly categorized into two groups: dense RRN (DRRN) and sparse RRN (SRRN) [6].

In the DRRN process, the mapping function (MF) between the subject and reference images can be established either linearly or nonlinearly using all the pixels from both images [7]. Histogram matching (HM) [8] is a widely employed DRRN method that effectively addresses radiometric inconsistencies between reference and subject images utilizing their respective histograms. Other frequently used DRRN methods, such as minimum–maximum (MM) [1] and mean–standard deviation (MS) [1], adopt global statistical parameters to establish a linear MF between the input images. DRRN methods are mostly used in image-mosaicking tasks because of their advantageous features, including time efficiency and the capability to handle large-sized image pairs [9]. However, when dealing with image pairs that include significant land cover/land use (LULC) changes, these methods may introduce noise structures and artifacts to the final results because of their equal treatment of all the pixels. SRRN methods, however, are specifically designed to handle radiometric distortions in such image pairs by extracting pseudo-invariant features (PIFs) and using them to establish a more precise MF [6,7,10,11].

In recent years, several SRRN methods [6,12,13,14,15,16,17] have been developed, each adopting a distinct approach for selecting PIFs from image pairs. The controlled-regression SRRN technique introduced by Elvidge et al. in 1995 [3], has appeared as a widely adopted and refined method for radiometric adjustment in the analysis of multitemporal images. In this method, PIFs/no-change pixels are identified using the scattergrams obtained from near-infrared bands through a controlled linear model. Another particularly powerful SRRN method is iteratively reweighted multivariate alteration detection (IRMAD), introduced by Canty and Nielsen [13], which has led to the development of many similar SRRN methods based on its principles. For example, Syariz et al. (2019) [18] presented a spectral-consistent RRN method for multitemporal Landsat 8 images, where PIFs were selected using the IRMAD method. In this method, a common radiometric level situated between image pairs was selected to reduce potential spectral distortions. Despite promising results, these methods primarily rely on iteratively identifying PIFs to re-estimate parameters for aligning images. A more advanced SRRN method was recently proposed by Chen et al. [10], in which PIFs in the shape of polygons were used to form an RRN model and generate reliable normalized images. Although these methods have shown promising results in RRN, they are limited to working with geo/coregistered image pairs and are therefore incompatible when unregistered input images need to be normalized [19].

To overcome the aforementioned limitation, keypoint-based RRN methods [19,20] have been developed that are robust to variations in scale, illumination, and viewpoints between subject and reference images. These methods typically operate in two steps. First, PIFs or true matches are extracted from image pairs through a feature detector/descriptor-matching process. Subsequently, an MF is created using these PIFs to generate a normalized image.

**Figure 1:**
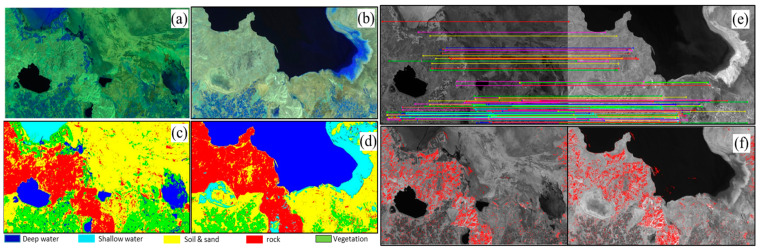
An example illustrating the limitations of keypoint-based and traditional SRRN methods in selecting PIFs from LCLU classes with varying spatial positions in coregistered reference and subject images. (**a**) Landsat 5 (TM) image acquired in September 2010 (subject image); (**b**) Landsat 7 (ETM+) image acquired in August 1999 (reference image); (**c**) LCLU map of the reference image; (**d**) LCLU map of the subject image; (**e**) selected PIFs from the reference and subject images based on the keypoint-based RRN method [21]; (**f**) selected PIFs from the reference and subject images based on the rule-based RRN [6].

Keypoint-based RRN methods effectively handle radiometric distortions in both registered and unregistered image pairs. Nevertheless, the efficacy of most SRRN methods and these approaches may be compromised when applied to image pairs with identical LCLU classes but located in different geographic areas because of factors such as seasonal fluctuations, climate variations, or other influencing elements. This arises as these methods often overlook these regions during their PIF selection, focusing primarily on spatially aligned PIFs or unchanged pixels to establish meaningful MFs between image pairs. For instance, Figure 1 highlights that both keypoint-based RRN and rule-based RRN methods [6] neglect deep and shallow water classes as potential regions for PIF selection, introducing the possibility of errors in the final results. This limitation becomes evident when these methods are used in scenarios with significant spatial LULC differences, potentially impacting the accuracy of the final RRN results.

On the other hand, DRRN methods are not limited by spatial constraints and can be considered to include all the LULCs in images during their normalization process. However, as mentioned before, using all the image pixels in their DRRN process may lead to suboptimal RRN results due to the existence of real change pixel pairs. This raises the question of whether an efficient RRN method can be devised to attain accuracy comparable to that of keypoint-based RRN methods while retaining the speed and spatial independence characteristics of DRRN, particularly in handling radiometric calibration for unregistered image pairs with varying LULCs in different locations.

To address this research question, we introduce the location-independent RRN (LIRRN) method, a novel approach designed to reduce radiometric distortions in bitemporal RS images, regardless of their coregistration status. LIRRN efficiently identifies PIFs from diverse ground surface brightnesses in image pairs by employing the dual approach of multithreshold segmentation and image content matching conducted band by band. This process starts with the bandwise multilevel segmentation of subject and reference images, categorizing pixels into dark, gray, and bright classes based on their gray values/digital numbers (DNs). Subsequently, a unique image-content-based matching strategy extracts PIFs from each class, considering the close similarity in DNs within the spectral range of the input images. This band-by-band matching ensures spatial independence and accurately represents the underlying content. By automatically extracting representative PIFs, this method facilitates precise radiometric normalization modeling in the final step of the normalization process. In the subsequent stage, the results of this method are fused with those of the keypoint-based method to enhance overall outcomes, particularly in scenarios requiring radiometric calibration for coregistered images.

This paper is organized as follows. Section 2 presents the proposed LIRRN method, detailing its key components and workflow. Section 3 describes the datasets used for evaluating the method, along with the evaluation metrics that were employed and a comprehensive analysis of the obtained results. Finally, Section 4 provides a summary of the paper’s findings and conclusions, as well as a discussion on potential avenues for future research and development in this field.

## 2. Materials and Methods

### 2.1. Methodology

Given bitemporal unregistered/coregistered RS images $MS^{S}=\left\{ms^{S}\left(i,j,k\right)|1\leq i\leq R,1\leq j\leq C,\;1\leq k\leq b\right\}$ and $MS^{R}=\left\{ms^{R}\left(i,j,k\right)|1\leq i\leq H,1\leq j\leq W,\;1\leq k\leq b\right\}$, defined respectively over the domains $\Omega^{s}=\left[1,R\right]\times \left[1,C\right]$ and $\Omega^{R}=\left[1,H\right]\times \left[1,W\right]$, in b spectral bands as subject and reference images, respectively, let us consider that these images were captured by either inter- or intra-sensors, depicting the same scene but acquired at different/same scales under varying illumination and atmospheric conditions.

**Figure 2:**
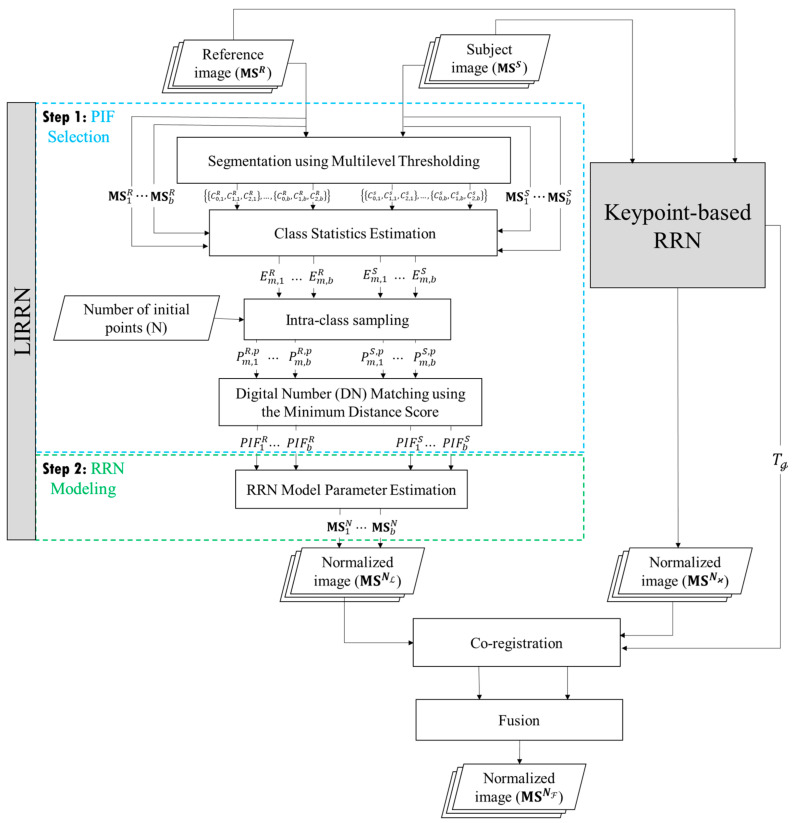
The flowchart of the proposed LIRRN method and its combination with the keypoint-based RRN for the radiometric calibration of unregistered/coregistered bitemporal multispectral image pairs.

The main goal of the LIRRN method is to efficiently generate a normalized subject image, $MS^{N_{L}}=\left\{ms^{N_{L}}\left(i,j,k\right)|1\leq i\leq R,1\leq j\leq C,\;1\leq k\leq b\right\}$, from $MS^{S}$ and $MS^{R}$ images regardless of their coregistration status. Figure 2 depicts the two primary steps of the LIRRN method: PIF selection (Section 2.1) and RRN modeling (Section 2.2). In the following scenario (Section 2.3), the image $MS^{N_{L}}$ is registered using the transformation matrix $T_{ℊ}$ from the keypoint-based RRN method and fused with its normalized image, $MS^{N_{\varkappa }}$, to generate a more accurate coregistered normalized image, $MS^{N_{F}}$.

#### 2.1.1. PIF Selection

This step aims to identify reliable and spatially independent PIFs from different gray levels of input images in a band-by-band manner. In detail, each band of $MS^{S}$ and $MS^{R}$ images is segmented into three classes (dark, gray, and bright) using the multilevel Otsu method, an extension of the classic Otsu thresholding technique [22]. In this method, a histogram, $H=\left\{f_{0},\;f_{1},\;\ldots ,\;f_{L-1}\right\}$, is constructed for each band of input images, where L represents the number of gray levels. The occurrence probability ($\rho_{i}$) for each gray level is determined by $\rho_{i}=\frac{f_{i}}{N_{n}}$, where $N_{n}$ denotes the total number of pixels in each image. Using threshold combinations, $\left(t_{1},\;t_{2}\right)$, where $0<t_{1}<t_{2}<L$, which were automatically detected, cumulative probabilities ($w_{m}$) and mean gray levels ($\mu_{m}$) for each class ($C_{m}\epsilon$ {$C_{0}$: dark, $C_{1}$: gray, and $C_{2}$: bright}) are calculated as follows:

(1) $$\begin{array}{c} w_{0}=\rho_{0}\\ w_{1}=\rho_{0}+\rho_{1}\\ w_{2}=\rho_{0}+\rho_{1}+\rho_{2} \end{array}$$

(2) $$\begin{array}{c} \mu_{0}=\frac{1}{w_{0}}\sum_{i=0}^{t_{1}-1}i\\ \mu_{1}=\frac{1}{w_{1}-w_{0}}\sum_{i=t_{1}}^{t_{2}-1}i\\ \mu_{2}=\frac{1}{w_{2}-w_{1}}\sum_{i=t_{2}}^{L-1}i \end{array}$$

The algorithm then iterates through the threshold combinations, $\left(t_{1},\;t_{2}\right)$, to maximize the between-class variance, $\sigma_{between}^{2}\left(t_{1},\;t_{2}\right)$, computed by considering the probabilities and mean gray levels for each class as follows:

(3) $$\sigma_{between}^{2}\left(t_{1},\;t_{2}\right)=w_{0}\left(t_{1}\right)\cdot \left(w_{1}\left(t_{1}\right)-w_{0}\left(t_{1}\right)\right)\cdot {\left(\mu_{1}\left(t_{1}\right)-\mu_{0}\left(t_{1}\right)\right)}^{2}+w_{1}\left(t_{2}\right)\cdot \left(w_{2}\left(t_{2}\right)-w_{1}\left(t_{2}\right)\right)\cdot {\left(\mu_{2}\left(t_{2}\right)-\mu_{1}\left(t_{2}\right)\right)}^{2}$$

The optimal threshold combination, $\left(t_{1},\;t_{2}\right)$, that maximizes the between-class variance is then selected.

Once each band of input images is segmented into the three aforementioned classes, PIFs are selected using the proposed image-content-based matching strategy. In this way, pixel values in the mth class of the kth band of $MS^{S}$ and $MS^{R}$ are first extracted and represented as $B_{m,k}^{S}$ and $B_{m,k}^{R}$, respectively. Class statistics, including the minimum (α), mean (β), and maximum (γ) of these pixel values, are then estimated and denoted as $E_{m,k}^{v}=[\alpha_{m,k}^{v},\;\beta_{m,k}^{v},\;\gamma_{m,k}^{v}],\;v:S,R$. Afterward, the differences between the extracted pixel values and estimated class statistics are obtained and sorted in ascending order, forming $\delta_{m,k}^{v,p}$ as follows:

(4) $$\delta_{m,k}^{v,p}=sort\;\left(\left|B_{m,k}^{v}-E_{m,k}^{v,p}\right|\right),\;v:S,R\;\mathrm{and}\;p:\;\alpha ,\;\beta ,\;\gamma$$

where $sort\left(.\right)$ is a function ascendingly rearranging the elements of vectors.

Subsequently, an initial subset, ${P_{0}}_{m,k}^{v,p}$, with N samples for each class in the kth band of each input image is selected based on the samples’ proximities to the estimated statistics as follows:

(5) $${P_{0}}_{m,k}^{v,p}=\left\{B_{m,k}^{v}|\delta_{m,k_{1}}^{v,p}\leq \left|B_{m,k}^{v}-E_{m,k}^{v,p}\right|\leq \delta_{m,k_{N}}^{v,p}\right\},\;v:S,R\;\mathrm{and}\;p:\;\alpha ,\;\beta ,\;\gamma$$

where $\delta_{m,k_{1}}^{v,p}$ and $\delta_{m,k_{N}}^{v,p}$ are the first and Nth elements of $\delta_{m,k}^{v,p}$, and N is also the number of the selected samples, which can be considered as being in the range [500, 10,000].

Randomly, 10% of these samples are chosen to form subsets $P_{m,k}^{v,p};\;v:S,R\;\mathrm{and}\;p:\;\alpha ,\;\beta ,\;\gamma$ for the mth class in the kth band of the $MS^{S}$ and $MS^{R}$ images.

To find corresponding DNs, a nearest-distance matching strategy is employed by computing pairwise Euclidean distances between the subsets $P_{m,k}^{S,p}$ and $P_{m,k}^{R,p}$, resulting in the matrix of pairwise distances, $D_{m,k}^{p}$. Sample pairs with minimum distances are then selected to create spectrally matched sets $M_{m,k}^{v,p};v:S,R$ and p: α, β, γ. Finally, concatenated PIFs for the kth band in each of $MS^{S}$ and $MS^{R}$ are formed by gathering samples within $M_{m,k}^{v,p}$ across all the statistics, resulting in vectors $O_{m,k}^{v};v:S,R$ for each class. Subsequently, vectors $O_{m,k}^{v}$ across all the classes are combined to generate the final set of concatenated PIFs, denoted as $PIF_{k}^{v};\;v:S,R$, for the kth band in each of $MS^{S}$ and $MS^{R}$.

#### 2.1.2. RRN Modeling

To establish the relationship between the $MS^{S}$ and $MS^{R}$ images and to generate the normalized image, $MS^{N_{L}}$, a linear regression was performed in a band-by-band way through the previously extracted PIFs in each band as follows:

(6) $$MS^{N_{L}}=\varphi_{0,k}+\varphi_{1,k}\left(MS^{S}\right)$$

where $\varphi_{0,k}$ and $\varphi_{1,k}$ are, respectively, the intercept and slope of the model, which can be obtained using the least-squares method as follows:

(7) $$\varphi_{1,k}=\frac{\sigma_{SR,k}^{2}}{\sigma_{S,k}^{2}}$$

(8) $$\varphi_{0,k}=\mu_{R,k}-\varphi_{1,k}\mu_{S,k}$$

where $\sigma_{S,k}^{2}$ refers to the variance in $PIF_{k}^{S}$, $\sigma_{SR,k}^{2}$ is the covariance between $PIF_{k}^{S}$ and $PIF_{k}^{R}$, and $\mu_{S,k}$ and $\mu_{R,k}$ signify the averages of $PIF_{k}^{S}$ and $PIF_{k}^{R}$, respectively.

For a clearer understanding, the step-by-step pseudocode of the proposed LIRRN method is presented in Algorithm 1.
**Algorithm 1** Location-independent Relative Radiometric Normalization (LIRRN)**Require:** $MS^{R}$ (Reference image), $MS^{S}$ (Subject image), *N* (Number of initial points, default = 1000)**Ensure:** $MS^{N_{L}}$ (Normalized subject image)  1:  **for**  k=1 to *b* **do**        ▷ Loop on the number of bands, i.e., *b*  2:   $C_{m}\in \left\{C_{0}\;\mathrm{dark},\;C_{1}\;\mathrm{gray},\;C_{2}\;\mathrm{bright}\right\}\leftarrow \mathrm{multilevelOtsu}\left(MS_{k}^{v}\right),\;\;v:S,\;R$  3:   **for**
m=0 to 2 **do**                       ▷ Loop on the number of classes  4:     $B_{m,k}^{v}\leftarrow \mathrm{extract}\;\mathrm{the}\;\mathrm{samples}\;\mathrm{of}\;\mathrm{class}\;m\;\mathrm{from}\;MS_{k}^{v},\;v:S,\;R$  5:     $E_{m,k}^{v}\leftarrow \left[\alpha_{m,k}^{v},\;\beta_{m,k}^{v},\;\gamma_{m,k}^{v}\right],\;\mathrm{where}\;E_{m,k}^{v}\;\mathrm{is}\;\mathrm{the}\;\mathrm{minimum}\;\left(\alpha \right),\;\mathrm{mean}\;\left(\beta \right),\;$ $\mathrm{and}\;\mathrm{maximum}\;\left(\gamma \right)\;\mathrm{of}\;B_{m,k}^{v}$  6:     $\delta_{m,k}^{\nu ,p}\leftarrow \mathrm{sort}\left(\left|B_{m,k}^{v}-E_{m,k}^{v,p}\right|\right)$,  v:S,R and p:α,β,γ               ▷ Equation (4)  7:    ${P_{0}}_{m,k}^{v,p}\leftarrow \left\{B_{m,k}^{v}|\delta_{m,k^{1}}^{v,p}\leq \left|B_{m,k}^{v}-E_{m,k}^{v,p}\right|\leq \delta_{m,k^{N}}^{v,p}\right\},\;v\;:S,R$ and p : α, β, γ       ▷ Equation (5)  8:     ${\;P}_{m,k}^{v,p}\leftarrow \mathrm{select}\;0.1\times N\;\mathrm{points}\;\mathrm{randomly}\;\mathrm{from}\;P_{0}^{m,k,v,p}$  9:     $\;D_{m,k}^{p}\leftarrow \mathrm{compute}\;\mathrm{pairwise}\;\mathrm{distances}\;\mathrm{between}\;P_{m,k}^{S,p}\;\mathrm{and}\;P_{m,k}^{R,p}$  10:      **for** r=1 to 0.1×N **do**       ▷  Loop on the number of randomly selected points  11:       $\eta_{m,k}^{p}\leftarrow \mathrm{compute}\;\mathrm{the}\;\mathrm{minimum}\;\mathrm{value}\;\mathrm{of}\;D_{m,k}^{p}$  12:       extract the row $\left(x_{m,k}^{p}\right)$ and $\left(y_{m,k}^{p}\right)$ of $\eta_{m,k}^{p}$ in $D_{m,k}^{p}$  13:       $\;M_{m,k}^{S,p}\left[r,1\right]\leftarrow P_{m,k}^{S,p}\left(x_{m,k}^{p}\right)$  14:       $\;M_{m,k}^{R,p}\left[r,1\right]\leftarrow P_{m,k}^{R,p}\left(y_{m,k}^{p}\right)$  15:       remove the element $D_{m,k}^{p}\left[x_{m,k}^{p},y_{m,k}^{p}\right]$ from $D_{m,k}^{p}$  16:     **end for**  17:   ${\;\mathrm{PIF}}_{k}^{v}\leftarrow U_{m}U_{p}M_{m,k}^{v,p}$                 ▷  Gather values from sets $M_{m,k}^{v,p}$  18:   **end for**  19:  $\mathrm{estimate}\;\mathrm{the}\;\mathrm{normalization}\;\mathrm{coefficients}\;\left\{\phi_{0,k},\phi_{1,k}\right\}$ using ${\mathrm{PIF}}_{k}^{S}$ and ${\mathrm{PIF}}_{k}^{R}$ in Equations (7) and (8)  20:   $\;MS_{k}^{N_{L}}\leftarrow \mathrm{apply}\;\mathrm{the}\;\mathrm{constructed}\;\mathrm{RRN}\;\mathrm{model}\;\left(\mathrm{Equation}\;\left(6\right)\right)\;on\;MS_{k}^{S}$  21:  **end for**  22:  **return**
$MS^{N_{L}}$


### 2.2. Fusion of LIRRN and Keypoint-Based RRN

In the context of the multitemporal image analysis, where the acquisition of a normalized coregistered image is pivotal, our approach involves the integration of an LIRRN-generated normalized image, $MS^{N_{L}}$, with the counterpart produced using a keypoint-based method, denoted as $MS^{N_{\varkappa }}$. In this scenario, the scale-invariant feature transform (SIFT) operates as the core for the keypoint-based RRN to generate the normalized image, $MS^{N_{\varkappa }}$, and geometric transformation matrix, $T_{ℊ}$ (i.e., generated herein using an affine transformation). The transformation matrix ($T_{ℊ}$) is then employed to coregister the LIRRN-generated normalized image ($MS^{N_{L}}$) with the normalized image, $MS^{N_{\varkappa }}$. Finally, they are fused using a weighted average to generate the fused normalized image ($MS^{N_{F}}$) as follows:

(9) $$MS^{N_{F}}=\frac{\omega_{N_{L}}MS^{N_{L}}+\omega_{N_{\varkappa }}MS^{N_{\varkappa }}}{\omega_{N_{L}}+\omega_{N_{\varkappa }}}$$

where $\omega_{N_{L}}$ and $\omega_{N_{\varkappa }}$ represent the weights assigned to each normalized image and are determined based on the reference image, which can be obtained as follows:

(10) $$\omega_{N_{L}}=\frac{1}{\left|MS^{R}-MS^{N_{L}}\right|}$$

(11) $$\omega_{N_{\varkappa }}=\frac{1}{\left|MS^{R}-MS^{N_{\varkappa }}\right|}$$

### 2.3. Data

**Figure 3:**
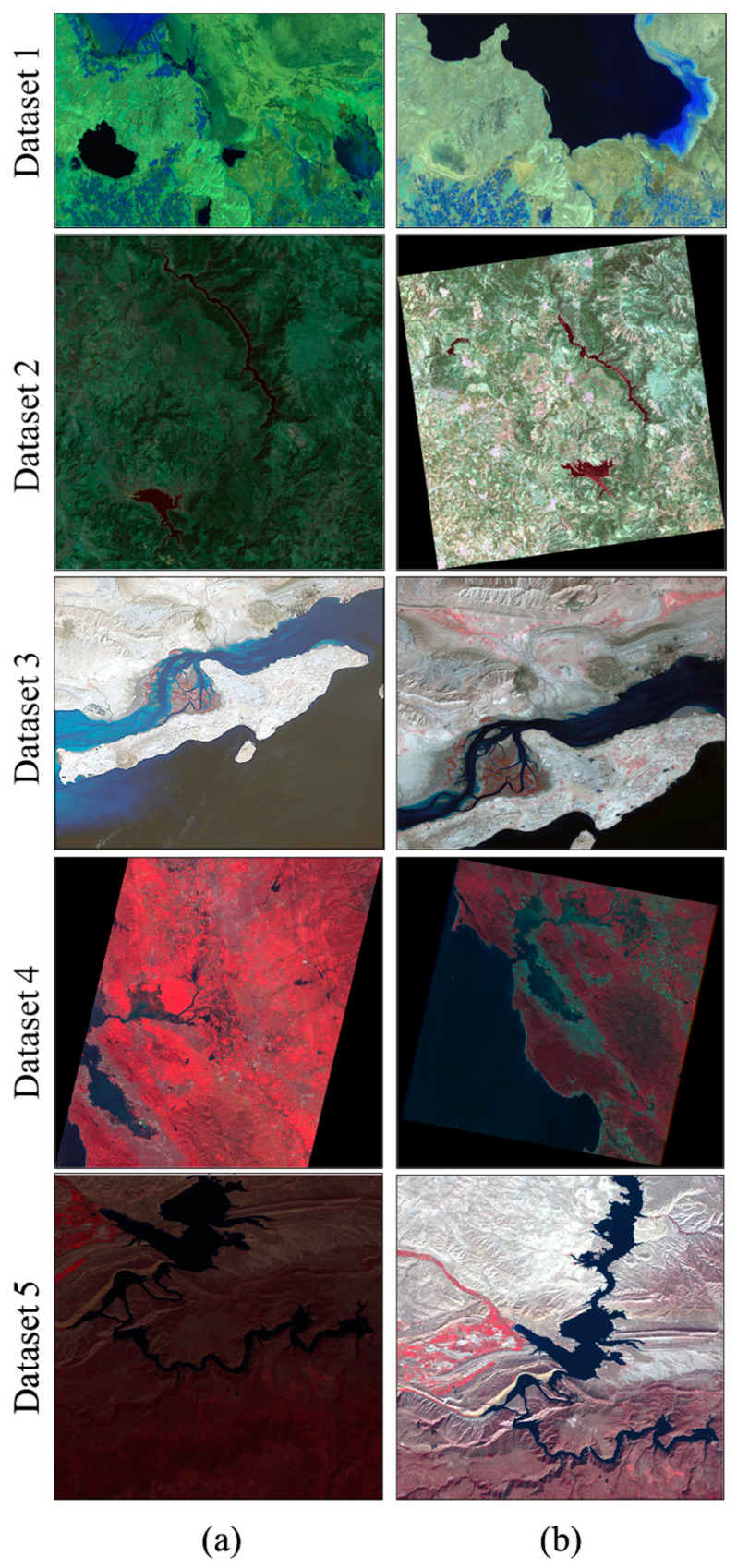
(**a**) Subject images and (**b**) reference images. Color–infrared composites (Green/Red/NIR) and (NIR/Red/Green) were employed respectively to show dataset 1 and datasets 4 and 5 and their results, whereas the normal color composite (Red/Green/Blue) was used to display datasets 2 and 3.

To conduct both quantitative and qualitative assessments, this study utilized five groups of unregistered bitemporal multispectral images captured by either the same/cross/different sensors under diverse imaging conditions (see Table 1 and Figure 3a,b). As can be seen from Table 1, datasets 4 and 5 include image pairs taken from the IRS (LISS IV) and Landsat 5/7 (TM/ETM+) sensors, exhibiting discrepancies in spatial resolutions and viewpoints. Datasets 1 and 3 were captured with identical spatial resolutions using Landsat cross sensors, whereas dataset 2 comprises subject and reference images taken by Landsat 7 (ETM+). It is essential to emphasize that the selected image pairs were not precisely coregistered, where all the subject images lacked geoinformation and were rotated or shifted to ensure a comprehensive evaluation of the effectiveness of the LIRRN method in handling non-georeferenced RS images.

### 2.4. Evaluation Criteria

The performance of the RRN methods was assessed by calculating the root-mean-square error (RMSE) for the overlapped area of the image pairs as follows:

(12) $$\mathrm{RMSE}=\sqrt{\frac{1}{N_{o}}\sum_{\iota =1}^{N_{0}}{\left(MS_{\iota }^{R}-MS_{\iota }^{N}\right)}^{2}}$$

where $N_{o}$ is the total number of pixels in the overlapped area. A low RMSE describes acceptable RRN results, while a high RMSE denotes worse results. It is worth noting that to calculate the RMSE, the reference and subject images were first co-registered with subpixel accuracy. Moreover, to evaluate the changes in the detection results, we used common metrics, like the false-alarm rate $\left(P_{FA}\right)$, the missed-alarm rate $\left(P_{MA}\right)$, total error (TE) rate, overall accuracy (OA), and F-score ($F_{S}$), which can be respectively obtained as follows:

(13) $$P_{MA}=\left(\frac{FN}{FN+TP}\right)\times 100\%$$

(14) $$P_{FA}=\left(\frac{FP}{FP+TN}\right)\times 100\%$$

(15) $$OA=\left(\frac{TP+TN}{TP+TN+FP+FN}\right)\times 100\%$$

(16) TE=100%−OA

(17) $$F_{S}=\frac{2\times \left(\frac{TP}{TP+FP}\right)\times \left(\frac{TP}{TP+FN}\right)}{\left(\frac{TP}{TP+FP}\right)+\left(\frac{TP}{TP+FN}\right)}\times 100\%$$

where true positive (TP) is the changed pixels that are accurately classified as changed regions, false positive (FP) is the background that is classified incorrectly as changed regions, true negative (TN) is the changed regions that are accurately classified as changed regions, and false negative (FN) is the changed regions that are incorrectly classified as the background.

## 3. Experimental Results

### 3.1. Experimental Setup

The LIRRN method was implemented using MATLAB (version 2018b) on a PC running Windows 10, with an Intel^®^ Core™ i5-6585R CPU (Intel, Santa Clara, CA, USA), clocked at 3.40 GHz, and 32.00 GB of RAM.

**Figure 4:**
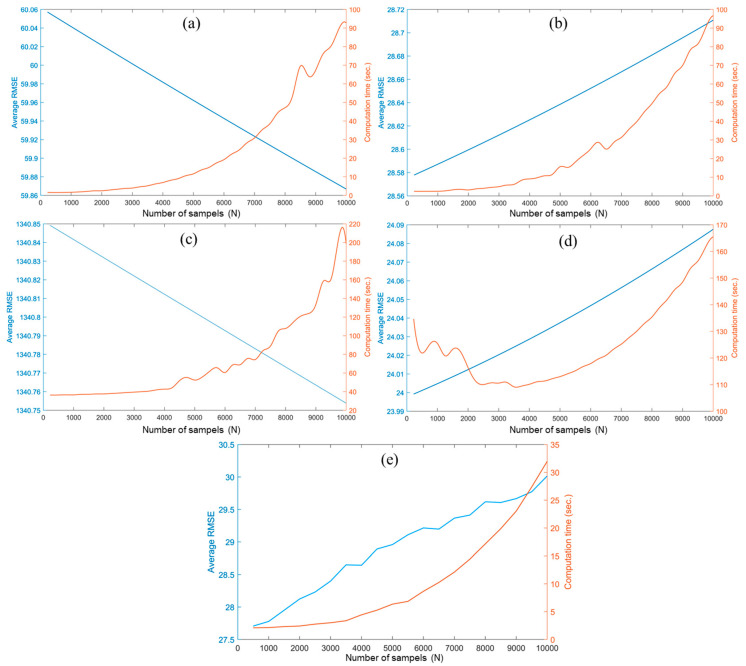
The average RMSE vs. the computational time of the proposed method for different numbers of samples (N) for datasets 1–5 (**a**–**e**), respectively. The blue and red lines refer to RMSE and computational time, respectively.

In our investigation, we examined the impact of the parameter N on the performance of the LIRRN method. To do so, we plotted the average RMSE and computational time against different values of N, ranging from 100 to 10,000 at intervals of 500, across all five datasets. These results are presented graphically in Figure 4.

Upon analyzing the plots presented in Figure 4, it became apparent that the average RMSE of the LIRRN method decreased with an increase in the number of samples for datasets 1 and 3. However, in the cases of datasets 2 and 4, it was observed that the average RMSE actually increased as the number of samples increased. Therefore, it shows that the effectiveness of the LIRRN algorithm is influenced by the choice of the parameter “N” across different datasets. These variations in the performance, whether they involve an increase or decrease, were relatively modest compared to the corresponding changes in the execution time. Across all the plots, it was evident that as the number of samples within the LIRRN process increased, there was a significant lengthening of the computational time, which makes LIRRN infeasible when dealing with large datasets, such as dataset 4. To address this concern, and according to our findings, we preferred to determine a value of N = 1000 in the LIRRN process to achieve a reasonable balance between accuracy and computational efficiency.

### 3.2. Comparative Results of the SRRN Methods

**Figure 5:**
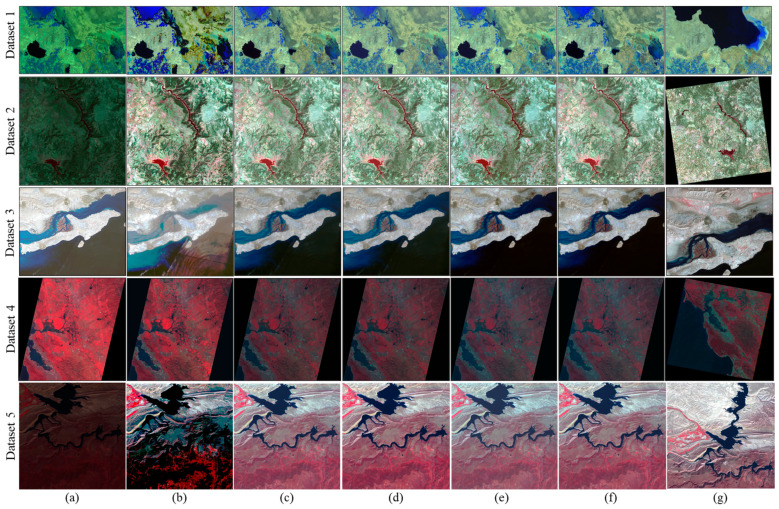
(**a**) Subject images and normalized subject images generated using (**b**) HM, (**c**) blockwise KAZE, (**d**) keypoint-based RRN, (**e**) LIRRN, and (**f**) fusion methods and (**g**) the reference image.

To assess the effectiveness of the LIRRN method, we conducted a comparative analysis against other relevant methods, including HM [8] and the blockwise KAZE method [19]. Additionally, the results for fusing the LIRRN with the keypoint-based approach were also included in our evaluation to further assess the efficacy of our fusion strategy. For this experiment, we compared both the accuracies and execution times, as seen in Table 2. Moreover, the normalized images produced using the considered techniques are presented in Figure 5 for a visual comparison.

The comparative results presented in Table 2 indicated that all the models effectively reduced radiometric distortions between image pairs, thus contributing to improved subject–image quality. However, it was noteworthy that the HM model, in certain datasets, yielded poor results, exhibiting an even worse performance than those of the raw subject images. Specifically, the proposed LIRRN method demonstrated superior performance over the HM, blockwise KAZE, and keypoint-based approaches in datasets 1–3, while the blockwise KAZE and keypoint-based methods showed better results for datasets 4 and 5, respectively. For example, the raw average RMSE was reduced by 12.59, 91.46, 10,517.84, 23.09, and 64.12 after employing the LIRRN method for the RRN of datasets 1–5, respectively. This indicates its potential to reduce radiometric distortion in unregistered cases or scenarios where the same LULC is observed in different locations of subject and reference images.

The integration of LIRRN with the keypoint-based approach yielded the most promising results across all the datasets, particularly emphasizing its effectiveness in datasets 3–5. For instance, after fusing LIRRN with the keypoint-based method, the average RMSE of the LIRRN method decreased by 11%, 8.5%, and 17.5% for datasets 3–5, respectively. Moreover, implementing the proposed fusion strategy led to a reduction in the average RMSE of the keypoint-based method by 5%, 3%, 15%, 0.1%, and 1% for datasets 1–5, respectively. This indicates that by employing a fusion strategy, we can achieve reasonable results that benefit from both methods, thereby ensuring the accuracy of the RRN when the radiometric calibration of registered cases is required.

The visual results also validate that all the considered methods, except for HM, successfully produced normalized subject images with exceptional color and brightness harmonization when compared to their corresponding reference images, as depicted in Figure 5. In further detail, there were no significant visual differences observed among the results of the blockwise KAZE, keypoint-based, proposed LIRRN, and fusion methods. However, the HM method exhibited artifacts and noise in the generated subject images, as evident in Figure 5b.

Additionally, the tone and contrast of the normalized images generated using HM were consistently higher than those of their corresponding reference images across all the cases. These characteristics can be attributed to the nature of the HM technique, which uses all the image pixels indiscriminately without considering time-invariant regions during processing. This approach leads to a bias toward changed region values, resulting in distortion within the RRN. However, despite its drawbacks, HM was found to be the most efficient RRN method in terms of its execution time, as it relies solely on the histograms of the image pairs as the core of its process.

On the other hand, the computational time of LIRRN was comparable to that of HM and, therefore, shorter than those of the keypoint-based method and the blockwise KAZE method. This makes LIRRN an efficient RRN method for handling large unregistered images. Moreover, the fusion of LIRRN with the keypoint-based method adds the computational time of the keypoint-based method to the execution time of the fusion approach. This is expected from the keypoint-based method, as it employs a matching process to extract PIFs and employs them for the simultaneous radiometry and coregistration of image pairs. Therefore, using the keypoint-based method and its fusion with the LIRRN method proves to be efficient when coregistered normalized images are required, despite the slightly longer computational time compared to those of the individual methods.

Table 2 also demonstrates that the performance of LIRRN is significantly superior to those of the other methods in datasets where image pairs have the same spatial resolution (datasets 1 and 2). This suggests that the performance of the LIRRN model is more affected when faced with datasets that include image pairs with different spatial resolutions. This issue is further discussed in Section 3.4.

### 3.3. The Impact of the Proposed Fusion-Based Strategy on Unsupervised Change Detection

To enable visual comparisons between the reference images and normalized subject images generated using the proposed fusion strategy method, we employed spectrum-based compressed change vector analysis (C2VA) [23]. Similar to the normalized images generated using the LIRRN method, the subject images were also coregistered with the reference images using the transformation matrix, $T_{ℊ}$. Subsequently, C2VA was utilized to depict changes between the subject and reference images, as well as between the normalized and fusion images and the reference images, in the 2D polar domain using magnitudes and orientations, as defined by Liu et al. in 2017 [23]. Finally, Otsu’s thresholding [22] was applied to the magnitudes of the C2VA to generate binary change maps from the inputs. To evaluate the change detection results, we have generated reliable ground-truth maps utilizing post-classification change detection techniques [24], followed by manual rectification to assign classes as changed or unchanged. In this experiment, the raw subject and reference images were regarded as the uncalibrated cases, whereas the normalized subject image and reference image were treated as the calibrated cases for the purposes of clarity and ease of explanation.

**Figure 6:**
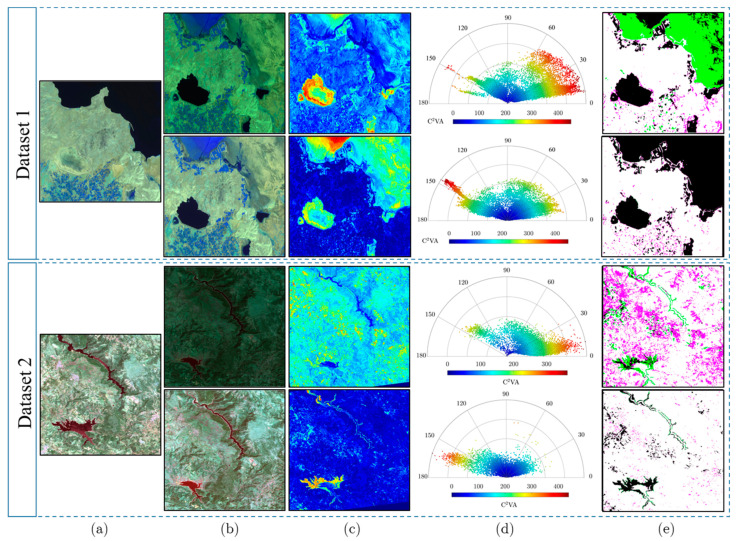
Comparison of C2VA-based change detection results before and after applying the proposed LIRRN method on a subset of datasets 1 and 2. (**a**) Reference images, (**b**) subject images (**top row**), and normalized subject images (**bottom row**); (**c**,**d**) C2VA and its 2D polar change representation domain, respectively, resulting from reference and subject images (**top row**) and reference and normalized subject images (**bottom row**); (**e**) change maps before (**top row**) and after (**bottom row**) applying LIRRN and overlaid with ground-truth data (black: changed; white: unchanged; magenta: false alarm; green: missed alarm).

Figure 6a–e illustrates the resulting normalized subject images generated using the fusion approach, along with the subject and reference images, C2VA magnitudes, and binary change maps superimposed on the ground-truth maps for a section of datasets 1 and 2.

The visual comparison presented in Figure 6 clearly indicated the significant impact of radiometric calibration on the accuracy of unsupervised change detection based on medium-resolution satellite images. Upon calibrating the datasets, the accuracy of the change detection significantly improved across all the datasets. Conversely, when utilizing uncalibrated datasets, a considerable number of missed alarms (depicted in green) and false detections (depicted in magenta) were observed in the generated change maps (see Figure 6e).

Moreover, the C2VA magnitudes derived from the calibrated images enable a clearer identification of the changed regions, thereby facilitating more straightforward thresholding for the generation of accurate change detection results. In contrast, magnitudes derived from uncalibrated images were significantly influenced by noise and anomalies. When derived from calibrated images, the 2D polar change representation domain exhibited a uniform distribution of unchanged regions characterized by low-magnitude values close to zero. This uniform distribution simplified the differentiation between changed and unchanged regions during the thresholding process, resulting in more precise change maps. Conversely, the non-uniform distribution of unchanged regions over zero in the 2D polar change representation domain, derived from uncalibrated images, posed a challenge in accurately distinguishing between changed and unchanged regions. These findings highlight the critical importance for using RNN in unsupervised change detection and confirm the fusion strategy’s superiority in this regard.

These results are supported by quantitative findings presented in Table 3. They demonstrate a significant decrease in $P_{FA}$, $P_{MA}$, and TE, while observing a considerable increase in OA and F_S_ scores following the utilization of calibrated datasets. This improvement is particularly evident when comparing the F_S_ score metrics, which exhibit an increase of over 40% after employing calibrated datasets with the LIRRN model. $P_{MA}$ is also notably decreased by ~50% subsequent to the radiometric calibration of the datasets.

### 3.4. The Impacts of the Angle and Scale on the Performance of the LIRRN

**Figure 7:**
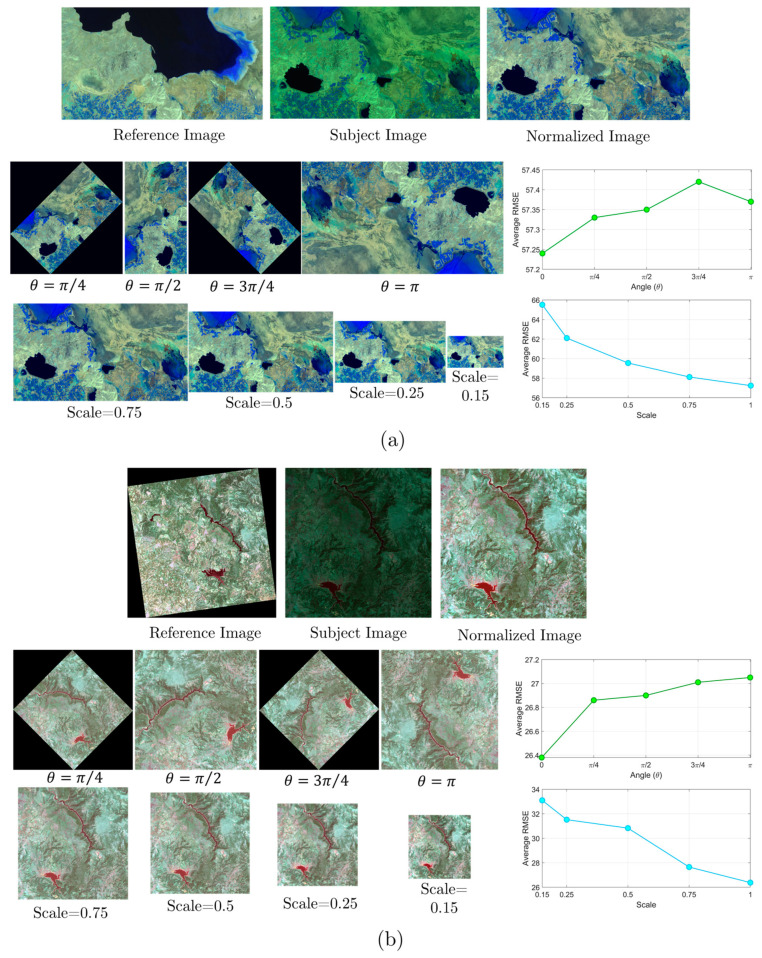
Comparative analysis of reference, subject, and normalized images generated using the LIRRN method at varied angles and scales applied to subject images from (**a**) dataset 1 and (**b**) dataset 2.

To examine the influence of varying angles on the LIRRN’s performance, subject images from datasets 1 and 2 were rotated in increments of π/4, ranging from 0 to π, and subsequently processed with LIRRN. Furthermore, to assess LIRRN’s effectiveness across different scales, subject images were subsampled at scales of 0.15, 0.25, 0.5, and 0.75 prior to the LIRRN application. The results of these investigations are shown in Figure 7.

As depicted in Figure 7, the normalized images generated using LIRRN at different angles and scales are well-aligned with the reference images, indicating the robustness of the LIRRN model to these distortions. Specifically, the plots in Figure 7 clearly show that the LIRRN method is more robust to angle variation compared to scale variation. For instance, the average RMSE remains relatively constant, with slight differences when the angle increases from 0 to π. In contrast, an increasing trend in the average RMSE is observed in the results of the LIRRN when reducing the resolution scale from 1 to 0.15.

## 4. Conclusions

In this paper, we proposed the LIRRN method, which can extract PIFs from both input images without relying on their coregistration status. The LIRRN method successfully extracted non-spatially aligned PIFs from various ground surfaces in image pairs by integrating the straightforward thresholding and proposed image content matching. This capability enabled the LIRNN method to successfully perform radiometric adjustment on unregistered image pairs and image pairs with LULC classes positioned differently. Moreover, the ability of the proposed LIRRN to generate registered normalized images was further boosted through fusion with the keypoint-based method.

To assess the effectiveness of the LIRRN method and its fusion, we tested them on five different RS datasets acquired using inter/intra-sensors. Our experimental results showed that LIRRN outperformed conventional techniques, such as the HM, keypoint-based RRN, and blockwise KAZE methods, in the radiometric adjustment of most considered datasets in terms of accuracy. Although the results of the LIRNN and keypoint-based and blockwise KAZE methods were similar, LIRNN was significantly faster and comparable to HM when the RNN of unregistered images was needed. This makes LIRNN an appropriate choice for online and near-online RS applications, where speed is critical. However, the performance of the LIRRN method has shown variability in some cases compared to the keypoint-based method. This variability may be attributed to the LIRRN method’s high focus on image content without considering spatial constraints during processing. This observation was further supported by the results obtained from the fusion of LIRRN with the keypoint-based method, where the proposed fusion method demonstrated more reliable and accurate results than when considering spatial and spectral constraints separately in the RRN process. However, the results of the fusion highly depend on the quality of the matching process embedded in the keypoint-based model. The results also indicated that the LIRRN method exhibited greater robustness against angle variations compared to changes in the resolution.

This study highlights the importance for integrating spatial constraints and image content into the normalization process to potentially enhance results. This suggests that incorporating the results from these factors within a suitable framework can lead to more reliable results and reduce distortions in target images. This highlights the significance of a holistic approach considering spatial and spectral constraints for improved performance in radiometric registration processes. The findings of our change analysis indicated that the proposed fusion approach successfully produced normalized images that can significantly enhance the quality of input images and improve the performance of change detection methods, like C2VA.

The limitations of the LIRRN model can be attributed to its dependency on parameters (N) and Otsu thresholding results. Therefore, investigating the use of learning-based models to automatically identify different parts of input images during the LIRRN process could be a promising avenue for future research. Additionally, integrating more advanced linear or non-linear machine-learning models into LIRRN has the potential to enhance its efficacy and yield even better results. Furthermore, using shape or land use/land cover (LULC) matching based on advanced deep-learning models could present an excellent opportunity to enhance the LIRRN method.

## Figures and Tables

**Table 1:** Characteristics of datasets.

Data	Ref./Sub	Satellite (Sensor)	Band Type	Resolution		Image Size (Pixels)	Date	Study Area
				Spatial (m)	Radiometric (Bits)			
# 1	$MS^{R}$	Landsat 7 (ETM+)	Blue; Green; Red; NIR *; SWIR * 1; SWIR 2	30	8	534 × 960	August 1999	West Azerbaijan, Iran
	$MS^{S}$	Landsat 5 (TM)				534 × 960	September 2010	
# 2	$MS^{S}$	Landsat 7 (ETM+)	Blue; Green; Red; NIR; SWIR 1; SWIR 2	30	8	582 × 574	May 2003	Cagliari, Italy
	$MS^{R}$					1131 × 1130	September 2002	
# 3	$MS^{S}$	Landsat 8 (OLI)	Coastal; Blue; Green; Red; NIR; SWIR 1; SWIR 2	30	12	3130 × 2405	June 2021	Qeshm Island, Iran
	$MS^{R}$	Landsat 9 (OLI-2)				2278 × 2292	November 2022	
# 4	$MS^{R}$	Landsat 7 (ETM+)	Green; Red; NIR; mSWIR 1	30	8	7871 × 7151	March 2002	San Francisco, CA, USA
	$MS^{S}$	IRS (LISS IV)		23.5		7883 × 7490	February 2022	
# 5	$MS^{R}$	Landsat 5 (TM)	Green; Red; NIR; SWIR 1	30	8	1000 × 1000	July 2009	Daggett County, UT, USA
	$MS^{S}$	IRS (LISS IV)		24		1000 × 1000	June 2020	

* NIR: near infrared; SWIR: shortwave infrared.

**Table 2:** Comparison of the RMSEs and computational times of the proposed LIRRN and fusion models and considered RRN methods for the datasets from 1 to 5.

Dataset #	Method	RMSE								Comp. Time (s)
		C/A	Blue	Green	Red	NIR	SWIR 1	SWIR 2	Avg.	
# 1	Raw	N/D	44.28	71.17	84.13	62.39	83.88	73.10	69.83	N/D
	HM	N/D	48.72	43.01	69.89	64.19	85.22	70.61	63.61	**1.08**
	Blockwise KAZE	N/D	37.74	44.22	66.39	**59.08**	77.35	**65.86**	58.44	37.1
	Keypoint-based RRN	N/D	43.70	48.41	69.90	59.78	78.04	67.23	61.18	24.82
	LIRRN	N/D	**37.34**	**41.85**	**59.41**	61.09	**77.22**	66.50	**57.24**	1.84
	Fusion	N/D	39.36	43.69	61.20	60.34	77.26	66.43	58.05	26.91
# 2	Raw	N/D	108.2	101.51	100.41	126.95	139.10	130.00	117.72	N/D
	HM	N/D	32.12	34.17	37.06	37.77	22.41	24.82	31.39	**1.02**
	Blockwise KAZE	N/D	30.02	31.63	34.49	32.51	14.61	19.61	27.14	31.74
	Keypoint-based RRN	N/D	28.71	33.63	36.04	32.52	13.62	18.95	27.25	20.33
	LIRRN	N/D	27.53	**31.24**	**33.71**	32.86	**13.53**	18.70	**26.26**	2.35
	Fusion	N/D	**27.43**	31.72	34.45	**32.65**	13.51	**18.54**	26.38	23.09
# 3	Raw	9151.3	10,031.94	11,779.02	12,232.78	13,582.19	14,545.61	13,249.57	12,081.77	N/D
	HM	2019.93	2034.29	2613.71	4011.93	5274.75	5195.34	4475.87	3660.83	**2.877**
	Blockwise KAZE	1191.36	1069.10	1107.65	1423.71	1854.64	2002.92	1788.16	1491.08	1210.21
	Keypoint-based RRN	1206.01	1103.10	1171.84	1506.03	1957.89	2128.69	1873.97	1563.93	890.64
	LIRRN	1223.28	1171.67	1536.92	1497.34	**1578.61**	1792.30	1578.01	1482.59	35.65
	Fusion	**1181.23**	**982.33**	**922.05**	**1204.12**	1662.05	**1726.35**	**1540.91**	**1317**	927.44
# 4	Raw	N/D	N/D	17.54	18.29	85.79	41.41	N/D	40.76	N/D
	HM	N/D	N/D	17.83	25.41	44.2	43.35	N/D	32.7	**6.03**
	Blockwise KAZE	N/D	N/D	11.86	17.36	18.24	19.18	N/D	16.66	5725.39
	Keypoint-based RRN	N/D	N/D	**10.66**	**17.01**	18.12	19.46	N/D	16.31	5558.84
	LIRRN	N/D	N/D	13.67	17.78	18.55	20.68	N/D	17.67	39.73
	Fusion	N/D	N/D	11.36	17.31	**18.09**	**17.95**	N/D	**16.18**	5590.61
# 5	Raw	N/D	N/D	98.15	89.59	89.25	90.43	N/D	91.86	N/D
	HM	N/D	N/D	56.70	49.27	75.18	63.33	N/D	61.12	**1.76**
	Blockwise KAZE	N/D	N/D	18.28	21.23	**25.74**	28.45	N/D	23.43	**61.84**
	Keypoint-based RRN	N/D	N/D	**17.04**	**20.27**	27.74	27.48	N/D	23.13	53.12
	LIRRN	N/D	N/D	24.06	26.19	27.53	33.18	N/D	27.74	**3.67**
	Fusion	N/D	N/D	17.66	20.49	25.92	**27.30**	N/D	**22.84**	58.11

**Bold numbers**: the best performance; N/D: no data; C/A: coastal aerosol.

**Table 3:** Change detection results obtained before and after applying the proposed LIRRN method on a subset of datasets 1 and 2.

Dataset #	RRN Status (× ^1^; ✓ ^2^)	$P_{MA}$ (%)	$P_{FA}$ (%)	TE (%)	OA (%)	F_S_
# 1	**×**	64.98	7.33	29.72	70.28	47.78
	✓	1.06	5.66	3.87	96.13	95.20
# 2	**×**	70.83	17.56	20.48	79.52	13.53
	✓	14.21	1.43	2.14	97.86	81.52

^1^ Before applying LIRRN; ^2^ After applying LIRRN.

## Data Availability

The code and some of the datasets are available at https://github.com/ArminMoghimi/LIRRN (accessed on 1 April 2023).
